# Efficacy of Intraoperative Widening of Surgical Resection Margins for Non-Palpable Breast Cancer: An Analysis of 771 Breast-Conserving Surgeries

**DOI:** 10.3390/cancers17233827

**Published:** 2025-11-28

**Authors:** Virginia Casati, Clémence Aellen, Elisa Ramazzotti, Camilla Favero, Marcel Kapahnke, Roberta Decio, Claudia Canonica, Nickolas Peradze, Simone Schiaffino, Diana Spinelli, Carola Mia Laura Catanese, Angela Lia Scarano, Ulrike Perriard, Meltem Yenigün, Maria Luisa Gasparri

**Affiliations:** 1 Department of Gynecology and Obstetrics, EOC, Ospedale Civico, 6900 Lugano, Switzerland; virginia.casati@eoc.ch (V.C.); elisa.ramazzotti@eoc.ch (E.R.); 2 Faculty of Biomedical Science, Università Della Svizzera Italiana (USI), 6900 Lugano, Switzerlandsimone.schiaffino@eoc.ch (S.S.); 3 Department of Gynecology and Obstetrics, Ente Ospedaliero Cantonale, Ospedale Regionale di Bellinzona e Valli, San Giovanni, 6500 Bellinzona, Switzerland; camilla.favero@eoc.ch (C.F.); marcel.kapahnke@eoc.ch (M.K.); claudia.canonica@eoc.ch (C.C.); 4 Centro di Senologia Della Svizzera Italiana (CSSI), Ospedale Regional di Lugano, 6900 Lugano, Switzerland; roberta.decio@eoc.ch (R.D.); nickolas.peradze@eoc.ch (N.P.); meltem.yeniguen@eoc.ch (M.Y.); 5 Istituto Imaging Della Svizzera Italiana (IIMSI), EOC, 6500 Bellinzona, Switzerland; diana.spinelli@eoc.ch (D.S.); carolamialaura.catanese@eoc.ch (C.M.L.C.); angelalia.scarano@eoc.ch (A.L.S.); 6 Centro di Radiologia e Senologia Luganese, 6900 Lugano, Switzerland; 7 Istituto Cantonale di Patologia (ICP), 6600 Locarno, Switzerland; ulrike.perriard@eoc.ch

**Keywords:** breast-conserving surgery, guidewire localization, intraoperative widening of surgical resection margins, margin status, tumor-free margins, non-palpable breast lesion

## Abstract

Background: The aim of the implementation of preoperative marking procedures in non-palpable breast cancer is to obtain a successful excision of the intended lesion with tumor-free resection margins while removing as little as possible of the surrounding healthy breast tissue to better preserve cosmetic integrity. Based on the intraoperative X-ray of the tumor specimen, intraoperative widening of selected surgical resection margins (IWSM) can be performed to decrease the positive margin rate. This study aims to evaluate the role of IWSM in breast-conserving surgery. Methods: All patients undergoing guidewire localized conservative surgery for non-palpable breast cancers were considered for the study. Patients, tumor characteristics, and pathological findings were collected. Tumor specimens undergoing intraoperative X-ray were considered for the analysis. The number of IWSM specimens, the IWSM specimens containing tumor cells at final histology, and the volume of IWSM specimens were calculated. Results: Data from 771 tumorectomies for non-palpable breast cancer performed between 2017 and 2023 at the Centro di Senologia della Svizzera Italiana (CSSI) were collected. A total of 751 tumorectomies with intraoperative X-ray assessment of the margin’s status were included. In 461/751 cases (61.4%), at least one IWSM specimen was obtained. At the final histopathology, in 354/461 IWSM specimens (76.8%), no tumor cells were reported, while they were reported in 107/461 (23.2%). Of these, 89/107 (83.2%) had overall negative margins, while 18/107 (16.8%) presented involved margins at the final histopathology. In 323/461 (70.1%) IWSM specimens, the pathological report provided three dimensions useful for volume calculation. The mean volume of these specimens was 9 ± 15 cm^3^. Conclusions: IWSM can help increase the efficacy of tumorectomies for non-palpable breast cancer lesions. However, in the majority of cases, healthy breast tissue is removed as well. Tumor and patient characteristics may help in the selection of cases for which IWSM reduces the positive margin rate and the number of unnecessary procedures.

## Simple Summary

The incidence of non-palpable breast cancer is rising. Breast-conserving surgery (BCS) followed by radiation therapy is the standard of care for most of the patients with breast cancer. The aim of breast-conserving surgery is to obtain a successful excision of the intended lesion with tumor-free resection margins. At the same time, goal of the procedure is to remove less healthy tissue as possible to obtain better cosmetic outcomes. Various techniques are available for lesion marking and intraoperative margin assessment. This study investigates the role of intraoperative widening of selected surgical resection margins (IWSM) in wire-guided BCS. IWSM is a simple, reproducible approach that may contribute to reducing positive margin rates in the surgical treatment of non-palpable breast tumors, with a minimal impact on the final resected volume.

## 1. Introduction

The number of early breast cancer diagnoses presenting as non-palpable breast lesions is increasing thanks to the improvement in and adherence to screening programs [1].

Upfront breast-conserving surgery (BCS) followed by radiation therapy is the gold standard for non-palpable breast cancer and early breast cancer with a favorable tumor-to-breast ratio, given the equally safe oncological profile and the better preservation of esthetic outcome when compared to mastectomy [2,3,4,5,6,7,8,9]. In most cases of ≥cT2 and/or clinical axillary involvement, and in selected cases of cT1c triple-negative and HER2-positive breast cancer, primary systemic treatment (PST) should be considered as an alternative to upfront surgery, despite an initial favorable tumor-to-breast ratio [5]. PST is often able to significantly reduce the size of the tumor, converting large tumors requiring mastectomy to small non-palpable lesions eligible for BCS [10].

The primary goals of BCS are the removal of the intended target lesion with negative margins (“no ink on tumor” for invasive disease; at least 2 mm for ductal carcinoma in situ (DCIS) [11] and the preservation of as much healthy tissue as possible. It is therefore mandatory to preoperatively localize non-palpable lesions.

Marking procedures can be performed in various ways, including wire-guided localization (WGL), radar reflector marking, magnetic or radioactive localization, radiofrequency identification tags (RFID), or ink-based localization [12,13]. Additional strategies can be adopted to intraoperatively confirm the efficacy of the tumor resection regardless of the type of marking procedure.

The intraoperative margin assessment can be performed through a frozen section with a preliminary evaluation of margins by the pathologist, as well as an intraoperative X-ray or ultrasound of the tumor specimen [14]. If the result of this preliminary analysis (obtained from the pathologist and/or radiological imaging) confirms the removal of the intended lesion but demonstrates a close tumor margin, a selective intraoperative widening of surgical resection margins (IWSM) can be performed to increase the chance of achieving a negative resection margin at the final histology analysis. Alternatively, some institutions directly perform cavity shaving after tumorectomy without a specific intraoperative margin analysis [8,14,15,16]. However, this approach seems to be more time consuming and less conservative, without the benefit of better surgical and oncological outcomes [14,16].

The aim of our study is to evaluate the efficacy of IWSM in wire-guided BCS after an intraoperative X-ray of the surgical specimen.

## 2. Materials and Methods

### 2.1. Study Design

A retrospective single-center analysis was performed.

### 2.2. Study Subjects

Patients undergoing conservative surgery with WGL for non-palpable malignant breast lesions and treated at the Centro di Senologia della Svizzera Italiana (CSSI) between January 2017 and December 2023 were included.

### 2.3. Study Setting

Both patients undergoing upfront conservative surgery for non-palpable breast cancer lesions as well as after PST were included. First diagnoses of breast cancer and local recurrences were considered for the analysis.

### 2.4. Study Variables and Procedures 

IWSM was defined as the intraoperative selected removal of additional tissue after tumor resection when intraoperative X-ray suggested a close or involved margin. Positive margins for invasive breast cancer were defined as “ink on tumor” (any invasive cancer or DCIS cells on ink) at the final histology analysis; positive margins for DCIS were defined as less than 2 mm of distance. IWSM was classified as useful when tumor cells were confirmed in the final histological analysis of the IWSM specimens while achieving negative margins. Conversely, IWSM was considered not useful in cases where no tumor cells were detected in the final pathology of the IWSM specimens or when involved margins were present in the final histological evaluation of the IWSM specimens. Patients and tumor characteristics, including menopausal status, PST use, history of radiotherapy on the affected breast, breast size, grading of the tumor, tumor size at pathological analysis, hormonal receptor status and HER2 expression, histological type of the tumor (ductal, lobular, or other), and DCIS or invasive breast cancer, as well as surgical and histological data, were retrieved from the clinical charts and then analyzed retrospectively.

### 2.5. Inclusion and Exclusion Criteria

Only patients undergoing intraoperative X-ray of the tumor specimen were included in the study. All surgical procedures included in the analysis were performed by a CSSI core team member. Exclusion criteria were benign non-palpable breast lesions and patients requiring mastectomy and surgical removal without localization or without intraoperative X-ray of the tumor specimen. Cases of tumorectomies that required oncoplastic procedures with the removal of additional tissue only for esthetic purposes were not included in the analysis.

### 2.6. Statistical Analyses

The total number of IWSM procedures completed, the number of IWSM specimens containing tumor cells at final histology, and the IWSM specimen volumes (when available) were calculated. A chi-squared test was performed to identify differences between patients without IWSM and those with at least one IWSM specimen based on intraoperative X-ray findings. The P-value was considered significant at ≤0.05. The IWSM specimen volumes were calculated when at least 3 dimensions were available. The primary outcome was the percentage of tumorectomies in which IWSM was useful. 

### 2.7. Ethical Considerations

The study was approved by the Ethics Committee in Switzerland (Rif. CE 4492, BASEC 2023-02124). The patients included in the analysis received non-objection letters according to Ethics Committee indication. 

## 3. Results

**Figure 1:**
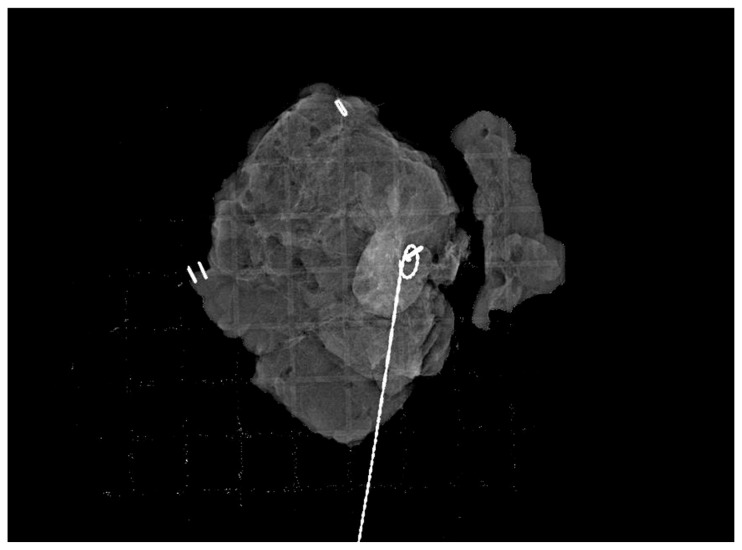
WGL tumorectomy specimen and its IWSM (scale: 1 cm = 1 grid square).

Of 1001 WGL tumorectomies, 230 could not be included because they either did not meet the criteria needed to be included in the study when a detailed clinical chart review was performed or because data related to the purpose of the study was missing. Nonetheless, we were able to analyze data from 771 non-palpable breast cancer lesions whose conservative surgeries had taken place between January 2017 and December 2023 at the CSSI. For 751/771 (97.4%) of these tumor specimens, an intraoperative X-ray for a preliminary indication on the status of the margins was performed, and they were included in the study. In 461/751 (61.4%) tumorectomies, the intraoperative X-ray findings led the surgeon to execute at least one IWSM (Figure 1). In the other 290/751 (38.6%) tumorectomies, no IWSM was performed. 

When comparing the patients and the tumor characteristics, no significant differences between tumorectomies with and without IWSM were found regarding menopausal status, PST use, history of radiotherapy on the affected breast, grading of the tumor, tumor size at pathological analysis, hormonal receptor status and Her2 expression of the tumor, histological type of the tumor (ductal, lobular, or other), and DCIS or invasive breast cancer. The only significant difference was found in breast size. Indeed, tumorectomies with IWSM were performed on breasts with cup sizes A–B in 191/461 (41.4%) of the cases and on breasts with cup sizes C–D in the remaining 270/461 (58.6%) cases. Respectively, tumorectomies without IWSM were performed on breasts with cup sizes A–B in 94/290 (32.4%) of the cases and on breasts with cup sizes C–D in the remaining 196/290 (67.6%) cases (*p*-value 0.0132) (Table 1). 

**Figure 2:**
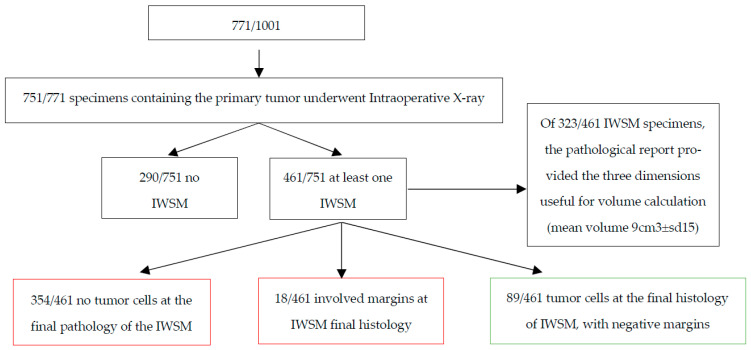
Flowchart showing overview of results. Green boxes show the “useful” IWSM and red shows the “not useful” IWSM.

Among all the tumorectomies with IWSM that were performed, 354/461 (76.8%) did not show tumor cells at the final pathology of the IWSM specimens. These were therefore considered “not useful”. On the other hand, histopathology confirmed the presence of tumor cells in the IWSM specimens in 107/461 (23.2%) of the cases. In 89/107 (83.2%) of them, the margins of the IWSM specimens were negative, and for 18/107 (16.8%) of them, the IWSM specimens’ final histopathology showed positive margins. If the margins were negative, we considered the surgery to be “useful”. If the surgery was negative, we considered it “not useful”, as the performed IWSM did not change the outcome (Figure 2).

In 38/751 (5.1%) cases, the primary goal of the surgery failed as the removal of the intended target lesion with negative margins was not achieved. These surgeries were therefore considered “ineffective”. In 20/751 (2.7%) cases, no IWSM was performed, while in 18/751 (2.4%) cases, at least one of the IWSM specimens had positive margins (Table 2).

In 11/751 (1.6%) cases, both the tumorectomy specimen and at least one of the IWSM specimens had positive margins. Interestingly, we also identified 7/751 (0.9%) cases in which the tumorectomies themselves had negative margins but at least one IWSM specimen had positive margins.

On the other hand, we found 89/751 (11.9%) cases in which the IWSM specimens contained tumor cells but with negative margins. In 18/751 (2.4%) cases, the tumor specimen itself had positive margins, and in 71/751 (9.5%) of them, the tumor specimen had negative margins at histology. In 12/751 (1.6%) cases, the tumor specimen had positive margins, and the IWSM specimens did not contain tumor cells at the final histology. All of these were considered effective surgeries, as well as the 270/751 (35.9%) cases in which the tumorectomy had negative margins and no IWSM was performed. In total, 371/751 (49.4%) of the surgeries were considered effective.

**Figure 3:**
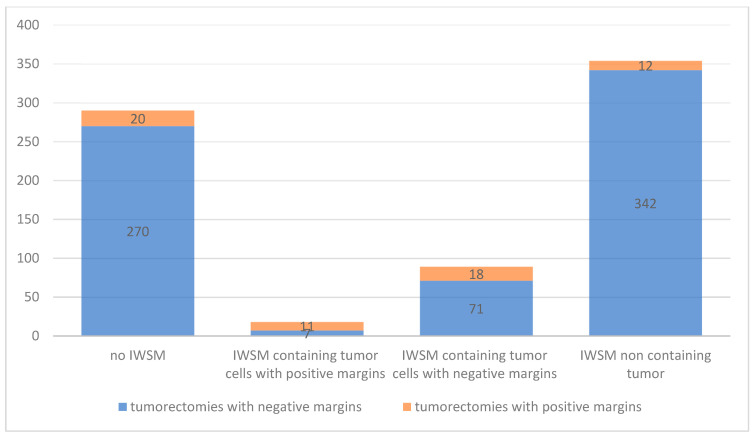
Tumorectomies with negative and positive margins based on IWSM.

In 342/751 (45.7%) cases, the tumor specimen had negative margins at the histology and IWSM was performed, which showed no trace of tumor. These cases were considered to be the “price to pay” to obtain negative margins (Figure 3).

Moreover, in 323/461 (70.1%) IWSM specimens, the pathological report provided the three dimensions useful for volume calculation. The mean volume of these specimens was 9 cm^3^ ± sd15.

## 4. Discussion

Several preoperative localization techniques have been developed to optimize the accuracy of tumor excision and ensure clear margins. Historically, the most well-known and commonly used method is WGL in which a metal wire is placed under ultrasound or mammography guidance [13,17,18]. The disadvantages of this technique are the non-negligible risk of dislocation of the wire, the need to insert the wire on the same day or the day before (not earlier), and discomfort for the patient [12,13]. Indeed, some more modern devices, such as probe-guided methods, have been developed in order to reduce the discomfort of patients and permitting the omission of an obligate procedure immediately before surgery [12,13].

An ongoing prospective trial promoted by the non-profit EUBREAST Network is currently evaluating different imaging-guided methods for localization of malignant breast lesions with a particular interest towards surgeon and radiologist satisfaction as well as patient tolerance [17].

In our case series, we decided to select a historical cohort of patients with non-palpable breast cancer undergoing BCS and used the conventional WGL to evaluate the efficacy of widening of the surgical margins, guided by the results of an intraoperative X-ray of the tumor specimen. 

The most important goal of BCS is to remove a lesion while minimizing the risk of positive margins as much as possible and reducing the amount of healthy tissue removed around the tumor site [11]. In the case of positive margins in the final histology report after breast-conserving surgery for invasive disease, reoperation for radicalization is generally considered mandatory [8,10,14,15,19]. In cases of DCIS where margins are close (<2 mm) but negative for an inked tumor specimen, a re-excision to achieve a 2 mm margin is advised and discussed on a case-by-case basis [8,14,15,19]. In our series, the positive margin rate is 5.1%, which is in line with data reported in the literature.

Several strategies can be implemented to reduce the incidence of positive margins [13], including intraoperative imaging of the specimen (such as ultrasound or X-ray) or intraoperative pathological assessment of the margins (such as frozen section and cavity shaving).

Intraoperative imaging has improved the assessment of resection margins and, most importantly, has helped reduce the number of re-excisions [14].

Intraoperative ultrasound (IOUS) offers an inexpensive way to evaluate the tumor both in vivo and ex vivo, providing a comprehensive evaluation throughout the surgical process and reducing overall costs and time requirements in the overall management of the patient [13]. However, it is a technique that requires radiological expertise since the results can vary depending on the pressure applied by the probe on the specimen, and surgeons and radiologists may therefore require appropriate training and experience [13]. Furthermore, this technique is not optimal for locating margins in tumors such as DCIS or lobular carcinoma [13,14]. Other limitations include limited penetration into deeper tissues, potentially causing lesions that are not adequately visualized to be missed, specifically small lesions; equipment availability; and potential for artifacts, particularly in fatty tissues [13].

An intraoperative X-ray of the specimen is convenient and efficient as it enables physicians to obtain two orthogonal views without compression, and since it can be performed directly near the operating room, it facilitates better identification of positive or close margins in a simple and fast way [5,14]. Also, in the case of a complete radiological response after PST, it can be helpful in confirming the accurate removal of the tumor bed through the localization of the clip [5]. Some authors have suggested that Digital Breast Tomosynthesis (DBT) enhanced precision when compared to an X-ray, and it is associated with a reduced incidence of re-excisions [14]. However, a randomized recent trial found no statistically significant difference between X-ray and DBT in regard to re-excision rates or the accuracy of margin assessment [6].

Micro-computed tomography (micro-CT) is another technique, offering superior spatial resolution. However, distinguishing tumors from the surrounding breast parenchyma becomes more challenging in dense breast tissue [14], and it is both time-consuming and expensive. Volumetric specimen imaging (VSI, 3D volume image created by algorithm from micro-CT) has shown superiority over X-ray and DBT, but it is still in the experimental stage [14].

Artificial intelligence (AI)-assisted mammography has been a subject of ongoing debate in recent meta-analyses, although AI on its own has not shown substantial superiority over traditional methods. In combination with standard mammography, it offers potential, particularly in improving diagnostic precision and standardizing image interpretation across different breast centers. This integration may help optimize clinical outcomes by enhancing margin assessments and minimizing variability [20].

In our series, all tumorectomies underwent intraoperative X-ray. When an X-ray of the specimen revealed a radiologically close or involved tumor margin, IWSM was performed at that specific site, offering the opportunity to obtain 101 effective cases with negative margins, with a “price to pay” of 342 when IWSM did not change the final result. Additionally, IWSM was significantly mostly adopted in breasts with cup sizes A–B as compared with C–D. We can speculatively justify this difference by considering that larger breasts undergo wider tumorectomies due to the greater amount of available tissue. In contrast, for smaller breasts, a more conservative approach is often adopted to preserve tissue, resulting in a smaller excision when compared to what would be performed in larger breasts.

As an alternative to the technique we used, a default cavity-wide margin extension can be performed. A shave margin constitutes tissue removal of approximately 2–3 mm in thickness from the tumorectomy cavity and should represent the size of the wall of the removed specimen [14]. Of note, it might be intuitive to consider that cavity shaving can be associated with the removal of a non-negligible percentage of “not useful” healthy tissue when compared to selected widening of a specific margin. However, this method can still be considered a valid option in specific circumstances like when alternative supporting tools are not available [8,15]. 

Some centers utilize frozen section analysis (FSA) to evaluate surgical margins intraoperatively. The margin of the specimen can be assessed intraoperatively through pathology, utilizing various techniques. Generally, the rate of re-excision for specimens with positive margins after intraoperative pathological analysis has been found to be around 10% according to recent studies [14,21]. This involves examining tissue samples immediately after surgical removal to assess the macroscopic tumor involvement of margins. This technique has been associated with low rates of re-excision and local recurrence (LR), providing surgeons with immediate feedback on margin status, which can help reduce the need for additional surgeries and improve oncological outcomes [6,7,22,23]. Although FSA is a validated technique and has been shown to be one of the most accurate for intraoperative margin assessment, its superiority in reducing positive margins compared to other methods has not been universally demonstrated. In fact, FSA’s accuracy, sensitivity, and specificity are comparable to those of other margin assessment techniques, and there remains a risk of false-negative results [6,7,15]. Furthermore, artifacts may be observed depending on the quantity of adipose tissue contained in the specimen, and the execution of this technique requires experienced pathologists close to or present in the operating room, which significantly increases the operative time [4,7,14,15,24]. However, the gold standard for margin assessment continues to be microscopic analysis of the surgical specimen, which is processed and sectioned using meticulous techniques beyond the scope of this discussion. This methodology necessitates a processing time of several days for accurate margin interpretation. Consequently, there is a risk that, in cases where positive margins are identified, the patient may require reoperation to obtain clear margins [7,14].

Regarding the average volume of re-excised tissue during IWSM, there is limited data available in the literature. Chen et al. reported an additional average volume of 12 cm^3^ of tissue removed by cavity shaving [22]. In our study, we observed an average of an additional 9 cm^3^ of tissue removed by IWSM depending on the number of IWSMs and the size of each additional specimen. As it was not possible to retrospectively obtain all data on the volumes of all tumorectomies at the time of the data analyses, we could not perform a quantitative analysis on the total amount of tissue (healthy and tumor) removed in the IWSM and non-IWSM tumorectomies. This is why we were unable to define whether the advantage of using IWSM is associated with an increased amount of tissue being removed despite it being the aim of our further analysis. In the meantime, we can speculatively state that, from an oncological standpoint, it is a safe procedure worth the cost of a small amount of healthy tissue removed, and although the preliminary analysis we conducted on the available IWSM volume data demonstrated an average volume of an additional 9 cm^3^ in the case of IWSM, we can still consider this procedure beneficial. 

### 4.1. Limitations

The limitations of this study are its retrospective design, along with the absence of some data, such as data on operative times that would have been a good indicator of cost-effectiveness, and a complete overview of the tissue removed that would give us the opportunity to calculate the optimal resection ratio.

### 4.2. Strengths

The strengths of this study are that it represents the first investigation of its kind, and it is supported by a large case series, providing comprehensive data. Additionally, despite the retrospective nature of the study, particular attention was paid to the purity of the sample. For instance, only procedures performed with the same localization method (guide wire) and by breast surgeons of the core team were included in order to accurately guarantee the same surgical procedure and the same level of surgical expertise in the cases we analyzed.

## 5. Conclusions

IWSM is a straightforward technique that can be implemented in any facility as it does not require specialized medical staff or complex equipment and is not associated with significant additional costs. Although it appears that in most cases, a small amount of additional healthy breast tissue is uselessly removed, IWSM can be helpful in decreasing the positive margin rate after wire-guided tumorectomies of non-palpable breast lesions. 

Further analyses are currently ongoing to demonstrate whether these results also apply to other localization methods, such as the probe-guided techniques, and whether these procedures affect oncological outcomes. Additionally, we are currently performing an accurate volume analysis investigating whether the performance of ISWM can affect cosmetic outcomes. 

## Figures and Tables

**Table 1:** Patients’ characteristics and IWSM.

	Total Number of Patients N = 751	Group 1 (At Least One IWSM Specimen) N = 461	Group 2 (No IWSM Specimen) N = 290	*p* Value (Group 1 vs. 2)
Menopausal status	593 (79.0%)	368 (79.8%)	225 (77.6%)	0.4633
PST use	97 (12.9%)	60 (13.0%)	37 (12.8%)	0.9187
History of RT on affected breast	16 (2.1%)	9 (1.9%)	7 (2.4%)	0.6698
Breast size: Cup A–B Cup C–D or larger	285 (37.9%) 466 (62.1%)	191 (41.4%) 270 (58.6%)	94 (32.4%) 196 (67.6%)	0.0132
Grading: G1–G2 G3	541(72.0%) 210 (28.0%)	332 (72.0%) 129 (28.0%)	209 (72.1%) 81 (27.9%)	0.9878
Tumor size at pathological analysis: pTis ≤ 2 cm or pT1 pTis > 2 cm or pT2/3	661 (88.0%) 90 (12.0%)	400 (86.8%) 61 (13.2%)	261 (90.0%) 29 (10.0%)	0.1842
Hormonal receptor status: Positive Negative	654 (87.1%) 97 (12.9%)	399 (86.6%) 62 (13.4%)	255 (87.9%) 35 (12.1%)	0.5830
Her2 expression: Negative Positive	651 (86.7%) 100 (13.3%)	394 (85.5%) 67 (14.5%)	257 (88.6%) 33 (11.4%)	0.2154
Histotype: Ductal Lobular Other	558 (74.3%) 100 (13.3%) 93 (12.4%)	342 (74.2%) 56 (12.1%) 63 (13.7%)	216 (74.5%) 44 (15.2%) 30 (10.3%)	0.9279 0.2349 0.1785
DCIS Invasive breast cancer	136 (18.1%) 615 (81.9%)	84 (18.2%) 377 (81.8%)	52 (17.9%) 238 (82.1%)	0.9199

**Table 2:** Final resection status of tumor and IWSM: + = positive margins, − = negative margins, / = N/A (as no IWSM was performed), * = overall positive margins.

Tumorectomies	IWSM	Final Resection Status at Histology (N = 751)
+	−	30
−	+	7 *
−	−	413
+	+	11 *
−	/	270
+	/	20 *

## Data Availability

The data presented in this study are available from the corresponding author upon reasonable request.

## Abbreviations

The following abbreviations are used in this manuscript:

AI	Artificial Intelligence
BCS	Breast-Conserving Surgery
CSSI	Centro di Senologia della Svizzera Italiana
DBT	Digital Breast Tomosynthesis
DCIS	Ductal Carcinoma In Situ
FSA	Frozen Section Analysis
HER2	Human Epidermal Growth Factor Receptor 2
IOUS	Intraoperative Ultrasound
IWSM	Intraoperative Widening of selected Surgical resection Margins
LR	Local Recurrence
Micro-CT	Micro-Computed Tomography
PST	Primary Systemic Therapy
RFID	Radiofrequency Identification
VSI	Volumetric Specimen Imaging
WGL	Wire-Guided Localization
